# There Is More to Mindfulness Than Emotion Regulation: A Study on Brain Structural Networks

**DOI:** 10.3389/fpsyg.2021.659403

**Published:** 2021-04-01

**Authors:** Sabina Baltruschat, Antonio Cándido, Antonio Maldonado, Carmen Verdejo-Lucas, Elvira Catena-Verdejo, Andrés Catena

**Affiliations:** ^1^Mind, Brain and Behavior Research Center (CIMCYC), University of Granada, Granada, Spain; ^2^Presentia, Mindfulness Training, Granada, Spain

**Keywords:** dispositional mindfulness, emotion regulation, structural networks, individual differences, MAAS, FFMQ

## Abstract

Dispositional mindfulness and emotion regulation are two psychological constructs closely interrelated, and both appear to improve with the long-term practice of mindfulness meditation. These constructs appear to be related to subcortical, prefrontal, and posterior brain areas involved in emotional processing, cognitive control, self-awareness, and mind wandering. However, no studies have yet discerned the neural basis of dispositional mindfulness that are minimally associated with emotion regulation. In the present study, we use a novel brain structural network analysis approach to study the relationship between structural networks and dispositional mindfulness, measured with two different and widely used instruments [Mindfulness Attention Awareness Scale (MAAS) and Five Facet Mindfulness Questionnaire (FFMQ)], taking into account the effect of emotion regulation difficulties. We observed a number of different brain regions associated with the different scales and dimensions. The total score of FFMQ and MAAS overlap with the bilateral parahippocampal and fusiform gyri. Additionally, MAAS scores were related to the bilateral hippocampus and the FFMQ total score to the right insula and bilateral amygdala. These results indicate that, depending on the instrument used, the characteristics measured could differ and could also involve different brain systems. However, it seems that brain areas related to emotional reactivity and semantic processing are generally related to Dispositional or trait mindfulness (DM), regardless of the instrument used.

Dispositional or trait mindfulness (DM) is the tendency to be aware of the present moment in a nonjudgmental and non-reactive manner. It can be seen as a resilience factor or as a risk factor for psychological health (Thompson et al., 2011). As a resilience factor, DM could act by preventing the emergence of a psychopathology, but as a risk factor (or lack of DM), it may increase the susceptibility to pathology and behavioral problems (Carpenter et al., 2019). Therefore, DM represents an important step toward understanding how mindfulness-based interventions could help to improve the efficiency of interventions on psychological health, and to disentangle the real benefits of intentional meditation from DM (Wheeler et al., 2016).

In fact, DM has been positively associated with psychological health and negatively with psychopathological symptoms (Tomlinson et al., 2018), including neuroticism (Hanley and Garland, 2017), substance abuse behavior (Karyadi et al., 2014), post-traumatic stress disorder (Boyd et al., 2018), self-esteem (Randal et al., 2015), anxiety and depression (Aldao et al., 2010; Hofmann et al., 2010), or pain (Zeidan et al., 2018). In spite of the considerable differences between these disorders, it is likely that these share one important factor in common, that is, the ability to regulate emotions (Farb et al., 2013; Tang et al., 2015). Emotion regulation refers to the process by which individuals intend to influence when, how, and for how long they experience and express emotions (Gross, 1998), and is thought to be at the core of various psychopathologies, such as alexithymia, panic attacks, anxiety, and abuse disorder, among others (Sheppes et al., 2015).

In this regard, mindfulness and emotion regulation are two psychological constructs that have shown a clear interrelationship, with the first serving as a regulator of the second (Chiesa et al., 2013; Roemer et al., 2015). Currently, it is thought that mindfulness practice can either give rise to a top-down control, in short-term users, or bottom-up control, in long-term users, which helps to regulate emotions (for a review, see Chiesa et al., 2013). The *a priori* tendency is to believe that being mindful underlies the ability to successfully cope with emotions, and that mindfulness enhances the strategies required to effectively regulate emotional reactions (Wielgosz et al., 2019). However, no research has been devoted to studying the cerebral basis of DM independently of emotional regulation, or which cerebral areas are related to DM and are only minimally dependent upon emotion regulation.

Emotional processing is underpinned by a set of brain regions encompassing those involved in emotional reactivity, such as the dorsal cingulate, insula, and amygdala, those involved in explicit emotional regulation, such as the dorsolateral and ventrolateral prefrontal cortex, supplementary motor area, and parietal cortex, and those involved in implicit emotional regulation, such as the ventral anterior cingulate (Etkin et al., 2015). Almost all these regions have also shown to be involved in mindfulness practice. For example, in a meta-analysis, Fox et al. (2014) suggest an effect of meditation practice on brain morphometry. They found differences between meditators and controls in the left anterior/mid cingulate cortex, right middle cingulate, midline anterior precuneus, left fusiform gyrus, right orbitofrontal cortex, left inferior temporal gyrus, left somatomotor cortices, and left anterior insular white matter.

Furthermore, Taren et al. (2013) demonstrated that two important subcortical areas—the amygdala and the caudate volumes—are involved in DM. The volume in the right amygdala and the caudate were negatively correlated with the Mindfulness Attention Awareness Scale (MAAS) scores (Feldman Barrett et al., 2007), one of the most used questionnaires used to measure trait mindfulness (Brown and Ryan, 2003). A similar result was obtained by (Lu et al., 2014), who found that volume in the right hippocampus/amygdala and anterior cingulate cortex (ACC) positively correlated with MAAS scores, but the left orbito-frontal cortex (OFC) and posterior cingulate volumes were negatively correlated. In a longitudinal study, (Friedel et al., 2015) demonstrated that cortical thinning in the anterior insula was correlated with DM, measured by MAAS. Given these findings, DM appears to be associated with ACC areas, which are related to cognitive control (Kerns et al., 2004), OFC, and amygdala/hippocampus, related to emotion regulation (Banks et al., 2007; Ulrich-Lai and Herman, 2009), the caudate nucleus, related to negative effect (Carretié et al., 2009), and the insula, related to interoceptive awareness and subjective experience (Gibson, 2019).

In a recent study, Shi et al. (2017) explored the cerebral basis of DM using brain volumes and cortical area and thickness. They found that MAAS was correlated with cortical volume of the right precuneus, and, that different dimensions of the Five Facet Mindfulness Questionnaire (FFMQ), another frequently used measure of DM (Baer et al., 2006), are related to volume, surface area, and thickness of fronto-parietal areas. Specifically, the describing dimension was observed to correlate with dorsolateral prefrontal cortex (BA 46) volume and surface area, as well as to inferior parietal lobule (BA 40) and superior PFC (BA 9) surface area. Furthermore, the non-judging dimension was related to the superior prefrontal surface (BA 10), while the non-reactivity dimension was related to the superior prefrontal thickness (BA 8). This study suggests that DM is associated with different brain structures according to the type of DM measure used (MAAS vs. FFMQ), and which feature of the brain structure is studied. Although DM dimensions seem to be associated to different cognitive control areas, more research is needed to draw conclusions.

Focusing on brain connectivity, Kong et al. (2016) demonstrated that regional synchronization of the brain correlated positively with MAAS scores at the right insula, left parahipocampal gyrus (PHG) and left OFC, but negatively with the right inferior frontal gyrus. Using resting state, Gartenschläger et al. (2017) have demonstrated that activity in the bilateral precuneus correlated positively, while that of the inferior frontal orbital gyrus (BA 47) and thalamus correlated negatively with MAAS scores. Moreover, Sharp et al. (2018) have suggested that right insula connectivity is increased by mindfulness meditation. Taken together, these findings suggest the involvement of the connectivity in and between structures related to self-awareness, such as the PHG (Chavoix and Insausti, 2017), precuneus areas (Felician et al., 2004), and the insula (Gibson, 2019), as well as the reduction of arousal, such as the thalamus (Fan et al., 2005).

Lim et al. (2018) have shown that global FFMQ scores correlated negatively with the connectivity between the dorsal attentional network (DAN) and the default mode network (DMN), which suggests that DM is related to the functional coupling of the brain areas responsible for attentional control and emotional regulation. In a similar vein, Harrison et al. (2019) have observed that higher DM was correlated with lower connectivity between the DMN nodes, but greater connectivity between the DMN and the somatosensory network. Parkinson et al. (2019) have observed that the FFMQ dimensions correlated positively with the activity in the nodes of those networks related to attentional control, interoceptive perception, and central executive functioning. The connectivity between functional networks dedicated to self-referential processing and mind wandering was decreased in individuals with higher DM scores. Thus, brain network results seem to confirm the relationship between DM and networks responsible for processing interoceptive stimuli, executive control, and regulating emotions.

In the light of this previous research, it is surprising that no studies have addressed the brain structural features that can account for DM when controlling for emotional regulation, since both constructs appear to have different but interrelated functions. To address this issue, in this study we used the most common measures of trait mindfulness, the FFMQ and the MAAS, and one common measure of emotional dysregulation, the DERS, together with brain structural networks. For the description of the brain structure, we selected a novel approach, the non-negative matrix factorization (NNMF) of gray matter volumes. This approach identifies brain structural networks and allows to find brain structures that consistently co-vary across participants in terms of dispositional mindfulness. In this way, the NNMF results from the decomposition of the whole brain gray matter into two matrices, one that indicates the weight of each voxel on each one of the networks (W), while the other matrix provides the weight of each participant on each network (H) (Sotiras et al., 2015). The H matrix is then used as a predictor in the analysis of measured individual differences. This approach has the advantage of building structural brain networks for the data sample, instead of a priori departing from an atlas-based partition, and has proven useful for classifying individuals (Varikuti et al., 2018). In this regard, our main outcomes are the scores of the FFMQ and the MAAS, while the predictors are all the brain structural networks plus age, gender, educational level, and DERS scores.

Since there is no literature on the relationship between mindfulness and brain structure, when areas related to emotional regulation are taken into account, this study is exploratory. However, we expect to find associations in brain areas similar to that found in previous literature, including areas associated to cognitive control and management of emotions, such as the prefrontal cortex, the insula, and the amygdala.

## Materials and Methods

### Sample Characteristics

We used structural MRI from 144 participants [50 women, mean age: 32.06 years old, range= (18, 68)]. All participants signed an informed consent form, were informed of their rights, and treated according to the Helsinki declaration (World Medical Association, 2013). All participants were paid for their participation in the study. The Ethics Committee of Human Research of the University of Granada approved this research (204/CEIH/2016). Five participants had missing data on the MAAS and were not analyzed when the MAAS scores were the outcome.

### Questionnaires

We used two mindfulness scales (MAAS and FFMQ) and one emotional regulation scale.

The Mindfulness Attention Awareness Scale (MAAS; Brown and Ryan, 2003); Spanish version by Soler Ribaudi et al. (2012) is a 15-item scale that measures attention to and awareness of the present moment within a unique factor. It has relatively good psychometric qualities (Cronbach's α = 0.89, accounting for 42.8% of variability).

The Five Facets of Mindfulness Questionnaire (FFMQ; Baer et al., 2006) is a 39-item questionnaire that assesses mindfulness, divided into five factors: Acting with awareness and focusing on activities in the moment (Awareness), labeling experiences (describing), non-judging of inner experiences and having a non-evaluative stance on thoughts and feelings (non-judging), non-reactivity to inner experience and allowing feelings and thoughts to come and go (non-reactivity), and attending to internal and internal experiences (observing). The version used here was that of (Cebolla et al., 2012), with good psychometric properties (Cronbach's α = 0.88, minimum Cronbach's α per scale = 0.80).

The Difficulties of Emotional Regulation scale (Gratz and Roemer, 2004), Spanish version by (Hervás and Jódar, 2008) has 39 items, organized into six factors: lack of emotional awareness (emotional awareness), lack of emotional clarity (emotional clarity), difficulty engaging in goal-directed behavior (goal-directed behavior), impulse control difficulties (Impulse control), unwillingness to accept certain emotional responses (non-acceptance), and lack of access to strategies for feeling better when distressed (strategies). The Spanish adaptation has good psychometric properties (Cronbach's α = 0.93, min Cronbach's α = 0.68).

### Brain Imaging Data Collection and Preprocessing

MRI scanning was conducted with a Siemens 3T Trio system equipped with a 32-channel head coil at the Mind, Brain, and Behavior Research Center (CIMCYC, Granada, Spain). Participants were instructed to avoid moving during the scan. To limit head motion, head restraint and foam padding around the head were used. A T1-weighted MPRAGE scan was obtained with a TR (repetition time) of 1,900 ms, TE (echo time) of 2.52 ms, and a flip angle of 9°. For each volume, 176 slices of 1 mm thickness were obtained, which provide whole brain coverage (voxel size = 1 × 1 × 1 mm; FOV = 256 mm; 256 × 256 data acquisition matrix).

The structural MRI scans were submitted to CAT12 toolbox (http://www.neuro.uni-jena.de/cat/) to obtain brain volumes, running under the umbrella of SPM12 (https://www.fil.ion.ucl.ac.uk/spm/software/spm12/), using default parameters. In essence, CAT12 corrects for bias inhomogeneity, segments into gray matter, white matter, and cerebrospinal fluid using the AMAP technique, and the images were spatially normalized using the Differmorphic Anatomical Registration through Exponentiated Lie algebra (DARTEL) algorithm. A mask with a threshold of 0.2 was used to avoid partial volume effects, which results in a total of 456,582 voxels.

### Non-negative Matrix Factorization

We used the non-negative matrix factorization (NNMF) described by Sotiras et al. (2015). NNMF factorizes a data matrix, the brain images of gray matter, into two non-negative matrices, H and W, representing the subject loading in each network (H, with size number of components by number of subjects), and the weight matrix (W, with size number of voxels by number of components). Following this, the H represents the weight in each structural network by each subject and is the primary data on which to build our predictive model. As NNMF identifies structural networks as a function of the interest of the user, not using an a priori parcellation of the brain, we explored solutions within the 10–200 range, with steps of 2. The reconstruction error (X-WH, where X represents the observed data) decreases as the number of networks increase, so we used the point of error stabilization, 100 networks. Therefore, the 100-network solution was used hereafter.

### Statistical Analysis

We used a multiple stepwise regression in which the outcomes were the dimensions of the FFMQ and the MAAS scores, and the predictors of interest were the structural networks of gray matter. The dimensions of the DERS, along with age, gender, and educational level were introduced as predictors of no interest. This allowed us to determine whether trait mindfulness can be predicted from the brain structure, discounting the prediction of the outcome that can be made from the dimensions of the emotional regulation questionnaire plus socio-demographics. A *p*-level of 0.01 was used for a variable to enter in the regression equation. Whole regression models were corrected for multiple comparisons using the Bonferroni approach. Note that positive or negative associations can be expected, but that a proper interpretation of the association is done using absolute values of the association.

## Results

### Questionnaires

The descriptive statistics of the study sample for the FFMQ, the MAAS, and the socio-demographic variables are displayed in Supplementary Table 1. The FDR-corrected correlations between the dimensions of the FFMQ, the MAAS score, and the socio-demographic factors can be found in Supplementary Table 2. Correlations are moderate with some non-significant values. Specifically, in the FFMQ, the observing dimension does not correlate with the awareness and non-judging dimensions; whilst non-judging does not correlate with the non-reactivity dimension. The MAAS score correlated with the awareness, non-judging and non-reactivity dimensions of the FFMQ. Gender correlated negatively with awareness and education level correlated negatively with the describing dimension.

FDR-corrected correlations between the dimensions of the DERS are displayed in Supplementary Table 3. Lack of emotional awareness only correlated with lack of emotional clarity, and lack of emotional clarity did not correlate with non-acceptance of emotional response. All the remaining correlations between DERS subscales were significant, while any of the socio-demographic variables showed any significant correlation.

Table 1 displays the FDR-corrected correlations between the scores of the three questionnaires. It should be noted that the observing dimension of the FFMQ correlated only with lack of emotional clarity and lack of emotional awareness dimensions of the DERS; the awareness and non-judging dimensions of the FFMQ did not correlate with lack of emotional awareness dimension of the DERS; and the non-acceptance dimension of the DERS did not correlate with the describing and non-reactivity dimensions of the FFMQ. It should also be noted that all correlations are negative, which indicates the similarity of the constructs since high DERS scores stand for emotion regulation difficulties.

**Table 1:** Correlations between FFMQ, MAAS, and DERS dimensions.

		**FFMQ**					**MAAS total**
		**Awareness**	**Describing**	**Non-judging**	**Non-reactivity**	**Observing**	
DERS	Emotional awareness	−0.153	−0.367	−0.021	−0.380	−0.359	−0.257
	Emotional clarity	−0.410	−0.554	−0.284	−0.445	−0.243	−0.391
	Goal–directed behavior	−0.440	−0.277	−0.305	−0.299	−0.134	−0.304
	Impulse control	−0.367	−0.332	−0.394	−0.366	−0.133	−0.356
	Non–acceptance	−0.290	−0.090	−0.501	−0.181	0.041	−0.308
	Strategies	−0.438	−0.326	−0.501	−0.417	−0.121	−0.406

*Shaded cells indicate significance after FDR correction*.

There are low to no associations between the dimensions of the FFMQ and the association with the total MAAS score. In fact, the largest correlation between the FFMQ dimensions is 0.50, which indicates that the dimensions are related to different processes and likely to different brain structures.

### Brain Structures

Table 2 displays the results observed for prediction of the FFMQ dimensions and the MAAS on the basis of brain structural components. The awareness dimension of the FFMQ can be predicted from the bilateral hippocampus, bilateral cerebellum 6, and limited access to emotional regulation strategies, *R*^2^ = 0.26, *p* < 0.05. The describing dimension of the FFMQ was only associated with the lack of emotional awareness and emotional clarity dimensions of the DERS, *R*^2^ = 0.38, *p* < 0.05. The non-judging of inner experience was accounted for by the bilateral cerebellum 4-5, bilateral precuneus/cuneus, right and left medial OFC, bilateral anterior fusiform gyrus, right and left superior frontal gyrus, and bilateral amygdala, along with the non-acceptance of emotional response and limited access to emotional regulation strategies dimensions of the DERS, *R*^2^ = 0.56, *p* < 0.05. The non-reactivity to inner experience was accounted for by bilateral anterior fusiform gyrus and bilateral inferior orbito- frontal cortex, in addition to the emotional awareness and impulse control dimensions of the DERS, and age, *R*^2^ = 0.31, *p* < 0.05. The observing dimension was accounted for by the bilateral cerebellum 9, left middle frontal and bilateral posterior fusiform gyri, along with age and the emotional awareness dimension of the DERS, *R*^2^ = 0.43, *p* < 0.05.

**Table 2:** Multiple stepwise regression results, regression coefficients, and *p*-values (in parentheses).

	**FFMQ**						
	**Awareness**	**Describing**	**Non-judging**	**Non-reactivity**	**Observing**	**Total**	**MAAS Total**
**Brain regions**							
L & R Cbe9					0.215 (0.002)		
L & R Hipp	0.372 (0.005)						0.072 (0.004)
L & R Cbe4-5			−0.396 (0.006)				
L & R Pcu/Cun			0.286 (0.009)				
L & R PHG						−1.170 (0.004)	−0.76 (0.002)
L & R Cbe 6	−0.373 (0.005)						
R MedOFC			−0.598 (0.007)				
L MidFG					0.456 (0.0002)		
L & R a Fus			−0.476 (0.001)	−0.425 (0.0001)		−1.340 (0.0006)	−0.055 (0.01)
R SupFG			0.475 (0.006)				
L & R InfOFC				0.326 (0.009)			
L MedOFC			1.072 (0.001)				
L SupFG			−1.254 (0.0001)				
L & R Amy			1.292 (0.0003)			2.845 (0.002)	
L & R p Fus					−0.661 (0.003)		
R p Ins						2.503 (0.0008)	

*Labels are from the Automatic Anatomic Labeling (AAL) atlas*.

*Amy, amygdala; Cbe, cerebellum; Fus, fusiform gyrus; Hipp, hippocampus; InfOFC, inferior orbito–frontal cortex; Ins, insula; MedOFC, medial orbito-frontal cortex; MidFG, middle frontal gyrus Pcu/Cun, precuneus/cuneus; PHG, parahipocampal gyrus, SupFG, superior frontal gyrus. a, anterior; p, posterior; L/R, left/right hemisphere*.

The total FFMQ score was accounted for by the bilateral parahippocampal gyrus, bilateral anterior fusiform gyrus, bilateral amygdala, and right posterior insula, along with age and the impulsive control and non-acceptance dimensions of the DERS, *R*^2^ = 0.62, *p* < 0.05.

The MAAS scores were predicted from the bilateral hippocampus, bilateral PHG and bilateral fusiform gyrus, along with age and the emotional awareness, impulse control, and non-acceptance dimensions of the DERS, *R*^2^ = 0.23, *p* < 0.05.

## Discussion

**Figure 1:**
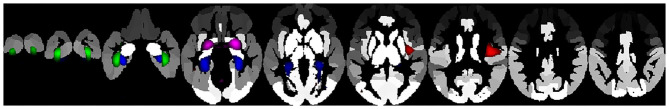
Structural networks accounting for significant variability in the FFMQ total score projected on the automated anatomic labeling (AAL) atlas. Green: bilateral fusiform gyrus, Blue: bilateral parahippocampal gyrus, Violet: amygdala; Red: posterior insula.

**Figure 2:**
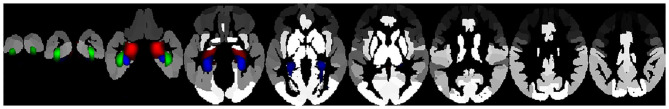
Structural networks accounting for significant variability in the MAAS score projected on the automated anatomic labeling (AAL) atlas. Green: bilateral fusiform gyrus, Blue: bilateral parahippocampal gyrus, Red: bilateral hippocampus.

In this study we aimed to determine the brain structural basis of DM using a non-negative matrix factorization approach, in which, instead of departing from atlas-based structural areas, covariance methods were used to form structural networks. We employed these structural networks to predict DM, as measured by the two most frequently used questionnaires, the FFMQ and the MAAS, while controlling for age, gender, educational level, and emotion regulation, as measured by dimensions of the DERS. Thus, our findings can be considered minimally dependent upon difficulties with emotion regulation. First, emotion regulation and DM are negatively related, so that highly mindful individuals tend to score lower on the DERS than those who are less mindful. This is in agreement with findings reported in the existing literature (Freudenthaler et al., 2017) and points to the possibility that DM could help to control emotional processing, although there is some residual variance in mindfulness that remains to be explained. Second, a number of mainly prefrontal, cerebellum, hippocampus, insula, and amygdala areas is necessary to predict the FFMQ dimensions and the FFMQ total scores, when discounting the DERS dimensions (Figure 1). Third, for the MAAS scores, the left and right hippocampus and bilateral parahippocampal cortex are significant predictors of the outcome, along with age, emotional awareness, impulse control, and non-acceptance of emotional responding (Figure 2).

For the FFMQ, the acting with awareness dimension is predicted from the bilateral hippocampus and bilateral cerebellum 6. The hippocampus has been implied in a variety of higher cognitive functions and is one of the first structures to show degeneration in certain forms of dementia, such as Alzheimer's disease (Evans et al., 2018; Ihara et al., 2018). As part of the memory circuit supporting narrative self-referential memories, it contributes essentially to emotion regulation (Hölzel et al., 2011). The hippocampus has a high concentration of corticosteroid receptors, which indicates that it is highly responsive to stress (Kim et al., 2007; McEwen et al., 2012) and a decrease in hippocampal volume is a risk factor for the development of stress-related disorders (Gilbertson et al., 2002). Larger hippocampal volumes have been repeatedly observed when comparing meditators with non-meditators (Luders et al., 2013; Joss et al., 2020), which also suggests that this structure is sensitive to enhanced mindfulness abilities. Further, the posterior lateral cerebellum is currently considered to be involved in cognition and emotion, in addition to motor tasks (D'Mello et al., 2020), particularly in embodied cognition (Guell et al., 2018), which takes into account the way we feel, think, and react (Wilson, 2002). In fact, cerebellar dysfunction can lead to cerebellar cognitive-affective syndrome, a syndrome that is characterized by deficits in executive function, spatial cognition, language, and, importantly, disinhibition and inappropriate behaviors in response to particular environmental situations (Schmahmann and Sherman, 1998; Schmahmann et al., 2007).

The non-judging dimension of the FFMQ is accounted for by a set of areas that encompass the bilateral cerebellum 4-5, the bilateral precuneus, bilateral anterior fusiform gyrus, bilateral amygdala, right and left medial OFC, and left and right superior frontal cortices. All these areas—with the exception of the bilateral amygdala, the fusiform gyrus, and the cerebellum—are part of the default mode network (DMN), the activity of which is related to situations in which attention is drawn away from external stimuli (Buckner et al., 2008), and has been shown to be involved in introspective thought, mind wandering, and rumination (Zhou et al., 2020). Moreover, the DMN has been related to brain psychopathology, including autism, ADHD, and depression (Buckner et al., 2008; Zhou et al., 2020). Certain parts of this network have been found to be sensitive to meditation practice, with long-term meditation being linked to reduced activation and functional connectivity of this network (Fox et al., 2014). The amygdala has also been associated with DM in a variety of studies (Fox et al., 2014), and appears to be involved in the control of emotional responding, in combination with other limbic structures (Taren et al., 2013; Creswell and Lindsay, 2014). Interestingly, an increase in amygdala volume is associated with an increased risk of stress-related pathologies (Shin et al., 2006).

The non-reactivity dimension of the FFMQ can be accounted for by the bilateral anterior fusiform gyrus and bilateral inferior OFC. The OFC has been reported to be involved in emotional processing and self-monitoring (Beer et al., 2006), and is thought to underlie the formation of self-referential representations of stimuli (Lin et al., 2018). Activity in the anterior fusiform gyrus has been found to correlate with non-reactivity in resting state conditions (Parkinson et al., 2019). Additionally, comparing long-term meditators to meditation-naïve participants, the former showed greater fusiform gyrus activity differences in the comparison of positive and neutral self-related (Lutz et al., 2016). This latter finding, together with the observation that this area is activated during various semantic tasks (both in non-verbal and verbal domains) including social concepts (Binney et al., 2016), indicates that it also plays an important role in semantic processing.

The observing dimension of the FFMQ can be accounted for by the bilateral cerebellum-9, bilateral posterior fusiform gyrus and right middle frontal cortex. The implication of posterior visual areas, such as the fusiform gyrus, in DM has been observed several times (Fox et al., 2016), but mainly when participants are required to respond to visual stimuli (Lutz et al., 2016). The mid prefrontal cortex is part of those areas that regulate affective responses (Ochsner and Gross, 2005) and could participate in attributions of individual emotional states (Barrett et al., 2007). The cerebellum-9 component is considered to be part of the dorsal attention network (Marek et al., 2018), which is devoted to the voluntary allocation of attention (Vossel et al., 2014).

The FFMQ total score is accounted for by the bilateral PHG, bilateral anterior fusiform gyrus, bilateral amygdala, and right posterior insula (Figure 1). The posterior insula is thought to be involved in interoceptive awareness (Allen et al., 2012; Farb et al., 2013), and has an increased connectivity with brain regions involved in reward and attention (Kirk et al., 2016). In addition, the connectivity between this area and the septal region was found to be enhanced in a mindfulness training group in comparison with a control group during the Ultimate Game (Kirk et al., 2016), which suggests that this structure is fundamental in the process of decision-making by switching attention to internal bodily experiences. (Kober et al., 2019), using meditation naïve participants, demonstrated that activity in the posterior insula, among other structures, decreases during a meditation in reaction to a heat stimulus. This finding indicates that activity in this area is modulated by mindfulness. The insular cortex is considered to be a hub of the connecting brain systems underlying cognitive, emotional, motivational, and sensory processes (Gogolla, 2017), involved in bodily awareness and self-awareness (Uddin et al., 2007; Hopkins et al., 2019). Since this structure has been found to be involved in top-down regulation in the processing of aversive, emotional, and bodily states, it has been proposed to underlie psychopathologies such as anxiety disorders or depression (Gehrlach et al., 2019). Moreover, it has been found that activity in the amygdala declines in response to positive stimuli and shows increased coupling with the prefrontal cortex in a group of individuals involved in a mindfulness-based program (Kral et al., 2018). The PHG has been reported to be involved in the processing of anger in a meditation context (Lee et al., 2017), which may be due to its dense connections with the amygdala, thus serving higher order functions such as self-control and emotion regulation (Stein et al., 2007). As part of the paralimbic regions, this structure has also been linked to psychopathy (Kiehl, 2006).

Similarly, MAAS scores were predicted by the bilateral hippocampus, PHG, and fusiform gyrus, overlapping with the areas accounting for FFMQ total scores, along with the hippocampus, which accounted for the awareness dimension of the FFMQ. This latter observation could be due to a similarity between the construct measured in the MAAS and the awareness dimension of the FFMQ, since both focus on the main characteristic of mindfulness, that is, attention and awareness of the present moment (Rau and Williams, 2016).

We have shown that DM is accounted for by a set of brain areas related to the control of emotional processing and self-regulation. Our results suggest that these areas support DM independently of emotional regulation. These structures belong to the default mode network (medial OFC, superior frontal cortex, rostral insula, and hippocampus), the visual network (precuneus, fusiform gyrus), the limbic network (inferior OFC), and the frontoparietal network (mid frontal cortex), in addition to the cerebellum and amygdala. This suggests that mindfulness abilities are distributed throughout the main areas of the cerebral hemispheres, and that it is important how we measure these abilities. If we consider DM to be a single psychological construct, measured by the MAAS and FFMQ total score, then this taps into two common areas, the PHG and fusiform gyrus. Thus, DM can generally be linked to the comprehension of semantics and the control of emotional responding. However, these two measures of DM differ in terms of three different structures (bilateral hippocampus for MAAS, and bilateral amygdala and right posterior insula for the FFMQ), and therefore do not seem to share a unified concept of mindfulness abilities. The fact that MAAS relies on the hippocampus may indicate that it depends more on the memory system, while FFMQ could potentially rely more on the emotional processing system (amygdala and right insula).

## Data Availability Statement

The raw data supporting the conclusions of this article will be made available by the authors, without undue reservation.

## Ethics Statement

The studies involving human participants were reviewed and approved by the Human Research Ethics Committee of the University of Granada (no. 204/CEIH/2016). The patients/participants provided their written informed consent to participate in this study.

## Author Contributions

ACá, ACa, AM, and SB designed the experiment. SB carried out the testing of participants. CV-L and EC-V designed and performed the intervention. ACa and SB analyzed the data and drafted the manuscript. All authors contributed to the critical revision of the manuscript and approved the final version.

## Conflict of Interest

CV and EC-V were employed by the company Presentia. The remaining authors declare that the research was conducted in the absence of any commercial or financial relationships that could be construed as a potential conflict of interest.
