# Supplementary material for: There Is More to Mindfulness Than Emotion Regulation: A Study on Brain Structural Networks
Source: Front Psychol. 2021 Apr 1;12:659403. doi: 10.3389/fpsyg.2021.659403 (PMC8046916; doi:10.3389/fpsyg.2021.659403)
Supplement: Supplementary file 1 [file Table_1.docx]

Supplementary Material

Supplementary Table 1. Mean scores of the questionnaires, and socio-demographic variables (mean, standard deviation and median)

| Questionnaire | Variable | Mean | SD | Median |
| --- | --- | --- | --- | --- |
| FFMQ | Acting with awareness | 27.465 | 5.403 | 28 |
|  | Describing | 28.271 | 5.939 | 29 |
|  | Non-judging of inner experience | 26.160 | 6.346 | 26 |
|  | Non-reactivity to inner experience | 22.361 | 4.309 | 22 |
|  | Observing | 27.292 | 5.305 | 28 |
| MAAS | Total score | 4.078 | 0.817 | 4.067 |
| DERS | Lack of emotional awareness | 15.868 | 4.650 | 15 |
|  | Lack of emotional clarity | 9.743 | 3.472 | 9 |
|  | Difficulties engaging goal-directed behavior | 13.354 | 4.481 | 12 |
|  | Impulse control difficulties | 11.715 | 4.541 | 11 |
|  | Non-acceptance of emotional response | 11.319 | 4.512 | 10 |
|  | Limited access to emotion regulation strategies | 14.799 | 5.434 | 13 |
| Demographic variables | Age | 32.063 | 13.503 | 24 |
|  | Educational level | 3.451 | 0.635 | 4 |

Supplementary Table 2. Correlations between the different dimensions of the FFMQ, the MAAS, and socio-demographic factors.

|  | FFMQ | | | | |  |  |  |  |
| --- | --- | --- | --- | --- | --- | --- | --- | --- | --- |
|  |  | Awareness | Describing | Non-Judging | Non-Reactivity | Observing | Age | Gender | Education |
| FFMQ | Awareness |  | 0.371 | 0.499 | 0.303 | 0.092 | 0.147 | -0.261 | 0.048 |
|  | Describing |  |  | 0.25 | 0.437 | 0.303 | 0.072 | 0.038 | 0.284 |
|  | Non-Judging |  |  |  | 0.234 | -0.173 | 0.016 | -0.136 | 0.077 |
|  | Non-Reactivity | |  |  |  | 0.382 | 0.1 | -0.116 | 0.208 |
|  | Observing |  |  |  |  |  | 0.132 | 0.021 | 0.177 |
| MAAS | MAAS Total | 0.553 | 0.229 | 0.277 | 0.264 | 0.194 | 0.03 | 0.044 | 0.373 |

Note: Shaded cells denote significance after FDR correction

Supplementary Table 3. Correlations between the different dimensions of the DERS and socio-demographic variables.

|  |  | DERS | | | | |  |  |  | |
| --- | --- | --- | --- | --- | --- | --- | --- | --- | --- | --- |
|  |  | E. Clarity | Goal-directed B. | Impulse control | Non-acceptance | Strategies | Age | Gender | Education |  |
| DERS | Emotional Awareness | 0.538 | 0.072 | 0.175 | -0.098 | 0.058 | 0.089 | -0.026 | -0.224 |  |
|  | Emotional Clarity |  | 0.455 | 0.440 | 0.170 | 0.523 | -0.223 | 0.008 | -0.156 |  |
|  | Goal-directed Behaviour |  |  | 0.565 | 0.359 | 0.683 | -0.147 | 0.141 | -0.086 |  |
|  | Impulse control |  |  |  | 0.404 | 0.647 | -0.165 | 0.046 | -0.147 |  |
|  | Non-acceptance |  |  |  |  | 0.591 | 0.059 | 0.013 | 0.025 |  |
|  | Strategies |  |  |  |  |  | -0.138 | 0.135 | -0.111 |  |

Note: Shaded cells denote significance after FDR correction
